# Automaticity of Attentional Bias to Threat in High and Low Worriers

**DOI:** 10.1007/s10608-016-9818-5

**Published:** 2016-11-19

**Authors:** Huw Goodwin, Claire Eagleson, Andrew Mathews, Jenny Yiend, Colette Hirsch

**Affiliations:** 1 Institute of Psychiatry, Psychology, and Neuroscience, King’s College London, London, UK; 2 University of California, Davis, Davis, CA USA

**Keywords:** Anxiety, Generalised anxiety disorder, Worry, Attention, Cognitive bias, Cognitive load

## Abstract

Individuals with high levels of worry are more likely than others to attend to possible threats, although the extent of top-down attentional control processes on this bias is unknown. We compared the performance of high (*n* = 26) and low worriers (*n* = 26) on a probe discrimination task designed to assess attention to threat cues, under cognitive load or no-load conditions. The expected difference between groups was confirmed, with high worriers being more likely to attend to threat cues than low worriers. Importantly however, there were no significant effects involving condition (cognitive load vs. no-load), nor any significant association with self-perceived attentional control ability. These results suggest that pathological worriers are more likely to attend to threat than are individuals with low levels of worry, regardless of task demands on limited cognitive control resources. This finding is consistent with the dominance of habitual bottom-up influences over top-down control processes in biased attention to threat.

## Introduction

Worry is experienced by most people to varying degrees (Borkovec 1994), but when excessive and uncontrollable, represents a key criterion of generalised anxiety disorder (GAD; Diagnostic and Statistical Manual 5th Edition; DSM-V; American Psychiatric Association 2013). Given the negative consequences of excessive worry, it is unclear why worriers continue to focus on negative thoughts, although some theories suggest that worry is a strategy for avoiding more aversive emotions (Borkovec et al. 2004), and that it is erroneously perceived as a helpful problem-solving strategy (Wells 1995). Such theories imply that worry is to some extent a voluntary process, involving deliberate attention to threats.

Attention is governed by two systems: an involuntary stimulus-driven (bottom-up) system that rapidly orients attention to salient information (Yantis 2000); and a more controlled goal-driven (top-down) system that modulates habitual attentional capture and serves to shift and maintain focus on task-relevant cues (Corbetta and Shulman 2002). The two systems interact and operate alongside each other to effectively focus and redirect attention so as to maintain safety and follow personally-relevant goals. However, some types of stimuli are particularly effective in capturing attention even when this interferes with current task goals. For example, threats often capture attention preferentially over neutral information, presumably representing an evolutionary adaptation to maintain survival (Öhman 1986). Biased attention to threat cues is particularly pronounced in anxious individuals (Yiend 2010) as demonstrated in studies using variants of the attention probe task (MacLeod et al. 1986). In these studies, anxious individuals have been found to respond more quickly to neutral targets in the same location as a threatening rather than a neutral cue (see Bar-Haim et al. 2007) implying an attentional bias towards threat (Becker et al. 2001; Bradley et al. 1999; Mathews and MacLeod 1985). Although anxiety and worry are related, with worry being seen as a cognitive component of anxiety; compared to studies focusing on anxiety, less is known about the specific relationship between worry and attention to threat.

Selective attention to threat has been postulated as a causal factor in Hirsch and Mathews’ (2012) cognitive model of pathological worry. According to this model, worry results from a combination of an automatic bottom-up bias with impaired top-down control processes, leading to intrusive negative thoughts. In support, Krebs et al. (2010) found that enhancing an attentional bias to threat words increased negative thought intrusions in the general population. Hayes et al. (2010) provided further support for this argument by demonstrating that training high worriers to attend *away* from threat led to a reduction in negative thought intrusions. In Hirsch and Mathews’ (2012) model, worry is then further maintained by an inability to voluntarily disengage from negative thoughts once they have captured attention. Other evidence suggests that worry itself can negatively impact attentional control by taking up working memory capacity, leaving fewer residual resources to shift attention to relevant task cues (Hayes et al. 2008; Leigh and Hirsch 2011; Stefanopoulou et al. 2014). The effect of worry in taking up attentional resources can be viewed as akin to an internal cognitive load, leading to a detriment in everyday task performance. Furthermore, imposition of additional cognitive load was found to have a greater deleterious effect on task performance among anxious individuals than non-anxious individuals (MacLeod and Donnellan 1993), presumably also due to the pre-emption of control resources by worry (Eysenck and Calvo 1992; Eysenck et al. 2007).

Although this evidence shows that both biased attention and impaired attentional control are characteristic of high worriers, it remains unclear whether or how they interact. One possibility is that the tendency to attend to severe threats is universal (Öhman 1986), but high worriers are less able to exert control over this tendency for milder threats, so that persisting attention to mild threat is really a consequence of poor control. Alternatively, even if attention to threat is typical of high but not low worriers, ineffective control might still prevent high worriers from countering the resulting bias (cf. Derryberry and Reed 2002). Both of these possibilities assume that high worriers typically make efforts (albeit ineffective) to avoid attending to threats; however, it could be that some do not attempt to exert such control, or instead focus their attention on threats in the belief that this is helpful (Wells 1995).

In summary, attentional bias to threat among individuals experiencing high levels of worry is fairly well established. Some cognitive theories (e.g. Hirsch and Mathews 2012) suggest that attentional bias may be modulated by top-down control processes, but there is no direct evidence that control efforts are deployed so as to either reduce or augment attentional bias. The present study was designed to provide such evidence by comparing attentional bias in high and low worriers, with and without an additional cognitive load. Manipulating available cognitive control resources in this way allowed investigation of whether control resources are normally deployed to reduce or augment attention to threat, or neither. That is, if control efforts are usually directed to countering attention to threat, imposition of a load should enhance bias, whereas if usually directed to attending threat, bias should be reduced under load. Alternatively, if control efforts are absent or ineffective, load should have little or no effect. Because threat bias has been found to be most apparent when primed by worry (Williams et al. 2014), the test of attention used here was preceded by a period of instructed worry. Finally, because attentional control may differ in a trait-like fashion across individuals, variations in self-reported control were also assessed to determine if attention to threat differed according to control ability (Derryberry and Reed 2002). The hypotheses to be investigated can be summarised as follows: (1) following worry, high worriers will attend more to threat cues than low worriers; (2) if control typically reduces or augments attention to threat, additional cognitive load will increase or decrease the assessed bias correspondingly, and (3) to the extent that efforts to control attention are effective, observed bias should vary according to self-reported control ability.

## Methods

### Design

After a brief period of instructed worry, both high and low worry participants performed a well-established attention probe task in which threat-neutral word pairs were displayed for 500 ms, followed by a target in the location of one of the words to be identified as rapidly as possible (MacLeod et al. 1986; for a review, see Yiend 2010). Selective attention to threat can be inferred from relative speeding of responses to targets replacing threat words. In the present study, this task was divided into two trial blocks of 216 trials each, presented in counterbalanced order, one without cognitive load and one in which participants were required to hold new sets of six digits in memory every 12 trials.

### Participants

A total of 66 non-clinical participants were initially recruited through online advertisements. To be included participants were aged between 18 and 60 years old, had English as their first language and indicated that they either worried a great deal or not much. They were first screened using the Penn State Worry Questionnaire (PSWQ; Meyer et al. 1990) and those scoring either ≥56 (high worriers) or ≤35 (low worriers) were invited for further testing. The score of 56 was chosen as being one *SD* below the mean score for individuals with GAD (Molina and Borkovec 1994), and the score of 35 as the mean value previously found for low worriers (Hayes et al. 2008). The PSWQ was re-administered at the testing session (within four weeks of screening) and nine individuals were excluded as they were no longer in the required range; a further four due to a high error rate (>33%) on the digit span task (indicating poor compliance with task); and one who had >10% errors (84% in no load condition) in the probe discrimination task. This left 26 high (women n = 22) and 26 low (women n = 20) worriers, with an average age of 25.6 years (*SD* = 5.43).

### Materials

#### Penn State Worry Questionnaire (PSWQ; Meyer et al. 1990)

The PSWQ is a 16-item self-report questionnaire measuring an individual’s trait level of worry. It includes questions such as “Many situations make me worry”, “Once I start worrying I cannot stop”, and “I have been a worrier all my life”. Participants respond using a scale of ‘1 = not at all typical of me’ to ‘5 = very typical of me’ and scores therefore can range from 16 to 80, with higher scores reflecting greater levels of trait worry. The measure has demonstrated good psychometric properties in clinical and non-clinical populations (Molina and Borkovec 1994) and had high levels of internal consistency in the current sample (Cronbach’s alpha = .98).

#### Generalised Anxiety Disorder-Questionnaire Version 4 (GAD-Q-IV; Newman et al. 2002)

The GAD-Q-IV is a self-report measure of diagnostic criteria for GAD using DSM-IV criteria, having good predictive properties for clinical diagnosis (Luterek et al. 2002). In the current study it showed high internal consistency (Cronbach’s alpha = .87).

#### Attentional Control Scale (ACS; Derryberry and Reed 2002)

The ACS is a 20-item self-report questionnaire that assesses attentional control ability. Example items include: “My concentration is good even if there is music in the room around me” and “After being interrupted or distracted, I can easily shift my attention back to what I was doing before”, rated on four-point Likert scales from ‘0 = never’ to ‘3 = always’. It has good psychometric properties (Muris et al. 2007) and was internally consistent in the current sample (Cronbach’s alpha of .91). Furthermore, the ACS has been shown to be positively correlated with objective cognitive measures of control (e.g. Judah et al. 2014).

#### State-Trait Anxiety Inventory-Trait Scale (STAI-T; Spielberger et al. 1970)

The trait scale of the STAI is a self-report scale of trait anxiety that includes 20 items such as: “I feel nervous and restless”, “I get in a state of tension or turmoil as I think over my recent concerns and interests”, rated on four-point Likert scales from ‘1 = almost never’ to ‘4 = always’. The STAI has demonstrated good psychometric properties in previous research (Barnes et al. 2002), and had high internal consistency in the current sample (Cronbach’s alpha of .96).

#### Beck Depression Inventory-II (BDI-II; Beck et al. 1996)

The BDI-II is a 21-item self-report questionnaire measuring depressive symptoms. Respondents are required to circle the statement (out of four) that best describes how they have been feeling for most of the time over the previous two weeks; for example, “I do not feel sad—0”, “I feel sad much of the time—1”, “I am sad all the time—2”, or “I am so sad or unhappy that I can’t stand it—3”. The BDI-II has shown good validity and reliability in previous samples (e.g., Storch et al. 2004), and was found to have high internal consistency in the current sample (Cronbach’s alpha = .94).

#### Emotion Rating Scales

Self-reported single item ratings of state worry, anxiety, depression, and happiness were recorded throughout the protocol to act as a manipulation check for the worry induction procedure (see below). Participants provided a state measure of worry (“How would you rate your worry in general at this moment?”; response range = 0–10; ‘10 = extremely worried’) before and after each worry induction task. In addition, visual analogue mood rating scales for anxiety, depression, and happiness were given before and after the worry induction (participants were instructed to indicate their level of the corresponding emotion on a 100 mm line, anchored with ‘not at all’ on the left hand end of the line and ‘extremely’ on the right hand end of the line).

### Attention Probe Task

Seventy two threat words were selected as representing typical worry concerns (e.g. “cancer”, “unloved”) identified from previous studies, and rated three or above by 20 independent assessors on a five point scale, ranging from ‘0 = not at all threatening’ to ‘5 = extremely threatening’. Neutral words were then chosen (using the English Lexicon Project website elexicon.wustl.edu) to match the threat words in length and frequency of use. Threat-neutral word pairs were presented in random order within the attention probe task, with each pair appearing three times in each experimental condition (i.e. with and without cognitive load). Word position within pairs was random, within the constraint that there were 54 trials in which each word type appeared in the upper or lower positions.

**Fig. 1:**
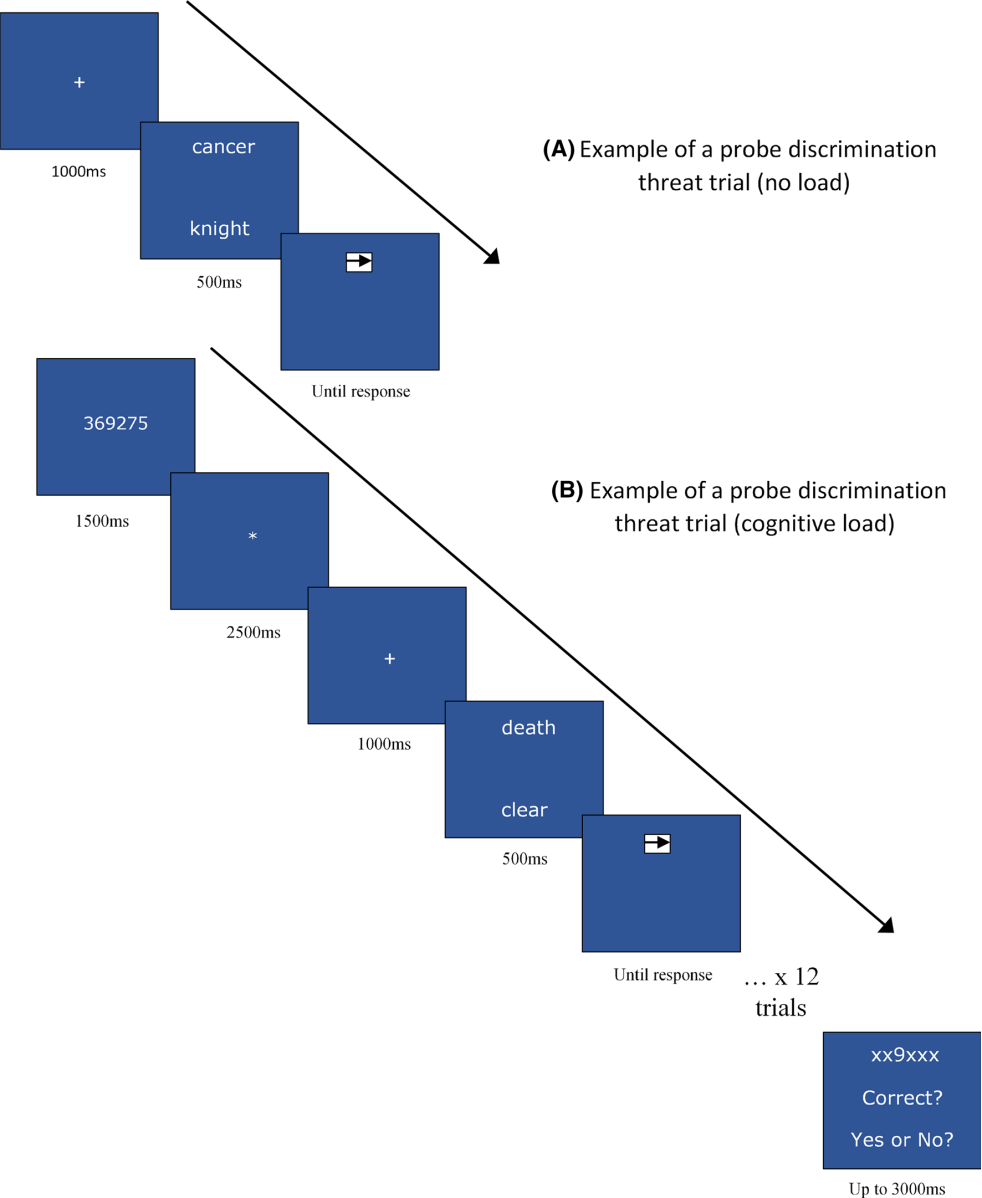
Example of probe discrimination task threat trial under no-load (**a**) and a probe discrimination task threat trial under cognitive load (**b**)

The task itself was carried out in a sound attenuated room on a laptop computer using the e-Prime program, with participants seated approximately 45 cm away from the screen. No-load trials began with a central white fixation cross (font size 18; on a navy background) for 1000 ms before being replaced by a threat-neutral word pair (font size 16, Microsoft Sans Serif), with one word above and one word below fixation, and a visual angle of 4.5 degrees between words. After 500 ms the word pair was replaced by an arrow (5 mm in length within a 5 mm × 5 mm white box), randomly in the position previously occupied by either the upper or lower word. Participants identified in which direction the arrow pointed by pressing marked keys as quickly as possible. Incorrect responses were signalled by a brief tone after which the fixation cross re-appeared (immediately or after 2500 ms if no response was detected) signalling the start of the next trial. There were 216 trials for each trial block (i.e. with or without cognitive load). A schematic representation of the task (with and without cognitive load) is shown in Fig. 1.

In the load condition, sequences of 12 trials began with the presentation of six to-be-remembered numbers (none with more than two numerically consecutive digits) for 1500 ms. This was followed by a star for 2500 ms before the fixation cross appeared (for 1000 ms) signalling the onset of 12 probed trials, same as for the no-load condition except for the requirement to remember the digit string. After completion of 12 trials (cf. Caparos and Linnell 2010), a string of five ‘x’s’ was displayed with one digit revealed (e.g., “XXXX7X”), and participants were required to indicate by pressing one of two marked keys whether or not the revealed digit was correct and located as in the to-be-remembered string. Participants had 3000 ms to make a response, in keeping with the work of Lavie et al. (2004), before receiving feedback (either a display of “correct” or “no response detected”, or an audible tone to signal errors). After 1500 ms, followed by a blank screen for 500 ms, a different set of six digits was presented and the procedure then continued as above for a total of 18 different number strings.

Previous research has suggested that attention to threat is more apparent following worry (Williams et al. 2014) and consequently the attention probe task was preceded by participants being asked to think of a worry that they had experienced in the previous week. Questions were used to elucidate the key aspects of the worry (e.g., “What would be bad about that?”, “What would that mean for you?”, “What worries/concerns you about that?”) after which participants were instructed to continue to worry about this topic for 5 min. This induction was repeated after the first block of the attention probe task (i.e. after 216 trials) using the same worry topic to ensure that the effect of the worry induction was present for the whole task.

### Procedure

On arrival participants were given an explanation of the study and any questions were answered before they signed a consent form. They then completed all questionnaires (PSWQ; GAD-Q-IV; ACS; STAI-T; BDI-II) followed by 12 practice trials on the attention probe task with neutral words only, and a further 18 trials with a cognitive load. They then continued on to the first worry induction, and completed the first block of the attention probe task, either in the cognitive load or no-load condition. The worry task was then repeated before proceeding to the second block with the alternative load condition. Throughout the attention task, participants were offered regular self-governed breaks when they could restart when ready. Finally, at the end of the session, they were debriefed, given an explanation of the experimental aims, and reimbursed for their participation (£15, equivalent to $23).

## Results

### Group Characteristics

Group characteristics were compared using independent sample *t*-tests, and means are shown in Table 1. Worriers were younger than low worriers (high worriers mean = 23.23, *SD* = 4.78, low worriers mean = 28, *SD* = 5.07*; t* (50) = 3.49, *p* = .001, *d* = 0.97), but Fisher’s exact test revealed no differences between groups in ethnicity (Caucasian vs. Black and Minority Ethnic Groups; *p* = .248). All participants were either in employment (67%) or in full-time education (33%), and 71% had completed a higher education course.

**Table 1:** Group characteristics and psychopathology measures

	High worriers (n = 26)	Low worriers (n = 26)
Gender (m/f)	4/22	6/20
Age	23.23 (4.78)^a^	28 (5.07)^a^
Ethnicity	White British = 17; Any other White background = 3; Mixed—White and Black Caribbean = 1; Mixed—White and Black African = 1; Mixed—White and Asian = 2; Indian = 1; Caribbean = 1	White British = 16; White Irish = 3; Any other White background = 5; Mixed—Any other mixed background = 1; African = 1

	Mean (SD)	Mean (SD)
PSWQ	64.15 (4.61)	27.23 (5.17)
STAI-T	52.77 (6.73)	30.69 (7.70)
BDI-II	15.12 (9.18)	2.73 (3.23)
ACS	46.35 (7.28)	59.42 (9.49)

*m/f* male/female, *PSWQ* Penn State worry Questionnaire, *STAI-T* State Trait Anxiety Inventory—Trait Scale, *BDI-II* Beck Depression Inventory-II, *ACS* Attentional Control Scale

^a^Mean (*SD*)

Nine participants (35%) in the high worrier group and none in the low group met criteria for GAD on the GAD-Q-IV. As expected, the groups differed on all other measures of psychopathology, with high worriers reporting higher scores than low worriers on the PSWQ, *t* (50) = 27.18, *p* < .001, *d* = 7.54, STAI-T, *t* (50) = 11.01, *p* < .001, *d* = 3.05, and BDI-II, *t* (31.1) = 6.49, *p* < .001, *d* = 1.8. In contrast, low worriers had higher scores on the ACS, *t* (50) = −5.58, *p* < .001, *d* = 1.55, indicating better reported attentional control.

### Emotion Ratings

Ratings of state worry across the whole sample significantly increased between pre- and post-worry inductions (Pre-worry *M* = 4.24, *SD* = 2.41; Post-worry *M* = 5.05, *SD* = .29). An additional paired *t* test comparing the change scores (for state worry) between pre- and post-induction after the first versus second induction was also performed. The results indicated that there was no significant difference between the two induction occasions in change of worry [t(51) = −.86, sig = .39, *M* change1 = .69, *SD* = 1.42; *M* change2 = .92, *SD* = 1.40].

As expected, an independent samples t test revealed that high worriers reported a significantly greater level of state worry than low worriers after the worry inductions [t(50) = 7.62, *p* < .001; High *M* = 7.00, *SD* = 1.41; Low *M* = 3.10, *SD* = 2.20]. However, a subsequent 2 (worry change 1 vs. worry change 2) × 2 (high vs. low worry) mixed ANOVA revealed that there were no significant effects of group [F(1,50) = 1.48, *p* = .23] and no significant group × time interactions [F(1,50) = 1.70, *p* = .20], indicating that the worry induction induced similar relative increases in worry in both low and high worriers. Additional emotion rating scales’ results can be seen in the footnote below.^1^

### Attention Probe Task

Prior to extracting median RTs, trials with errors (1.9%), or with response latencies <200 or >2000 ms (0.12%) were excluded, as per previous research (Bradley et al. 1999). An ANOVA of trial errors, with two within-participant factors (word valence: threat vs. neutral; condition: cognitive load vs. no-load) and one between-participant factor (group: high worriers vs. low worriers) revealed no main effects of valence, *F*(1,50) = 1.42, *p* = .239, $\eta_{p}^{2} = .03$, load condition, *F*(1,50) = 1.53, *p* = .222, $\eta_{p}^{2} = .03$, or group, *F*(1,50) = 1.12, *p* = .294, $\eta_{p}^{2} = .02$).

For the primary analysis of latencies, a threat bias index was calculated (cf. Bradley et al. 1999; MacLeod and Mathews 1988; MacLeod et al. 2007). This index was calculated by subtracting the median RTs on threat trials (i.e., when the probe replaced a threat word) from the median RTs on neutral trials (i.e., when the probe replaced a neutral word) for each participant, so that positive values represent attention bias *towards* and negative values *away* from threat. The mean RTs and threat bias indices for high and low worriers can be seen in Table 2. A mixed-design ANOVA was performed on this data with group (high vs. low worriers) as the between-participant factor and condition (cognitive load vs. no-load) as the within-participant factor. There was a main effect of group, *F*(1, 50) = 4.24, *p* = .045, $\eta_{p}^{2} = .08$ [high worriers mean = 4.01 (12.52) vs. low worriers mean = −2.56 (10.41)]. However, there was no significant main effect of condition (cognitive load vs. no-load; *F*(1, 50) = .10, *p* = .749, $\eta_{p}^{2} = .002$) nor a condition × group interaction, *F*(1, 50) = .01, *p* = .913, $\eta_{p}^{2} < .001$. Consequently, these results failed to provide any evidence that cognitive load increased or decreased attentional bias to threat, since the pre-emption of cognitive control resources by load failed to show significant effects in either direction.

**Table 2:** Probe discrimination task mean (SD) latencies and threat bias indices

	High worriers (*n* = 26)			Low worriers (*n* = 26)		
	No-load	Cognitive load	Total	No-load	Cognitive load	Total
Mean RT	556.49 (46.58)	568.39 (51.50)	562.44 (46.86)	567.54 (55.78)	569.58 (64.30)	568.56 (58.11)
TBI	3.34 (20.49)	4.69 (14.67)	4.01 (12.52)	−2.89 (14.79)	−2.23 (13.73)	−2.56 (10.41)

*RT* reaction time, *TBI* threat bias index

To investigate the possible role of self-reported attentional control ability (cf. Derryberry and Reed 2002), a stepwise regression analysis was conducted with threat bias index as the dependent variable, and group or attention control score (ACS) as predictors. Entering group in the first step confirmed the previous finding that threat bias index was predicted by high versus low worry, R^2^ = .078, F(50) = 4.24, *p* < .05. Adding ACS scores in the second step did not significantly add to predictive variance, R^2^ change = .012, F(49) = 0.67, n.s.; nor did adding the group by ACS interaction in step three, R^2^ change = .001, F(48) = .053, n.s. These results indicate that, although ACS scores were signicantly lower in high than in low worry groups, they did not predict threat bias, either overall, or differentially within worry groups. Consistent with the findings of no significant effects due to cognitive load, these results suggest that variations in self-reported attentional control do not significantly influence attentional bias to threat, at least at the 500 ms stimulus onset asynchony (SOA) used here.

## Discussion

The current study was designed to investigate attentional bias to threat in high worriers, and specifically whether this bias is modified by top-down control, or is relatively automatic. As expected, we found that high worriers showed more evidence of attention to threat cues than did a comparison group of low worriers. Although this effect was small, it confirmed a similar effect previously observed in high worriers using the same task (Williams et al. 2014), demonstrating its reliability. We also found that high worriers reported less attentional control ability than low worriers on a self-report questionnaire (ACS) that has previously been found to correlate with objective measures of control (Judah et al. 2014). This result is consistent with other experimental findings that high worriers are relatively impaired in working memory tasks, particularly when worrying (e.g. Hayes et al. 2008). However, level of reported control was not predictive of attention to threat, suggesting that reduced ability to control attention does not play an important causal role in attentional bias. Of most importance for present purposes, imposition of an additional cognitive load failed to significantly decrease the extent of biased attention to threat (numerically, bias was non-significantly *larger* under load). These findings strongly suggest that attention to threat in high worriers is relatively automatic, at least in the sense that voluntary control efforts are either lacking, or if deployed have no observable impact.

Of course, absence of evidence for control is not necessarily evidence of its absence, so that we cannot conclude that attentional bias to threat can never be influenced by top-down control. The SOA of 500 ms was adopted partly because previous evidence has suggested that control over attention can occur within this interval, at least in those with high ACS scores (e.g. Derryberry and Reed 2002), but it may be that use of longer intervals would have revealed clearer evidence of interference with control. Also, different degrees or types of cognitive manipulations may block attentional bias more effectively; for example by matching working memory load to each individual’s capacity, or using a perceptual rather than conceptual load cf.; Lavie et al. 2004; Pessoa et al. 2002).

Consequently, one limitation of the present study is that we cannot be certain that the load imposed took up sufficient cognitive resources to prevent any control efforts. The lack of a main effect of load on overall response latencies could be interpreted as support for this possibility. However, the relatively automatic nature of this task *per se* (e.g. responding to a leftwards arrow by pressing a left hand key) presumably demands very little resources compared with those required to control selective attention (e.g. specifically to threat cues). Furthermore, the load used here is typical of those found in many previous studies to take up significant attentional control resources (e.g. Lavie et al. 2004).

Nonetheless, effective voluntary control presumably depends on the *intentio*n to attend or avoid as well as having sufficient cognitive resources to succeed in doing so. The lack of any clear influence of cognitive load could thus reflect lack of intent or motivation to control attention to threat cues, rather than necessarily being due to impaired control *ability*. Either way, the lack of significant effects on bias due either to load or reported level of attentional control provides evidence that high worriers do not typically exert effective control over the capture of attention by threat content, at least within a relatively early time frame. In the model proposed by Hirsch and Mathews (2012), early attention to intrusive threatening thoughts acts as a trigger for worry episodes, so that failure to control attention to threat cues can contribute to pathological worry.

Whether or not high worriers can control their attentional biases has implications for the treatment of pathological worry. If they can, but do not implement the cognitive effort required to do so, then therapy should be directed at increasing that effort; for example, by countering beliefs that may underlie lack of effort, such as that worry is helpful or cannot be prevented (Wells 1995). If, in contrast, high worriers have impaired control of attentional biases then the use of training methods to enhance control resources would be indicated although research so far has shown rather mixed effectiveness (e.g., Harrison et al. 2013). Alternatively, the present results could imply that attention to threat is a relatively automatic process, and one that is difficult to oppose by voluntary efforts alone. If so, this would support alternative approaches bypassing the need for effortful control over attention to threat, for example by increasing the availability of competing information via repeated practice in attending to benign cues or meanings (Hayes et al. 2010; cognitive bias modification, see Hertel and Mathews 2011).

The current study’s findings may have implications for the development of treatments for people with high levels of worry. However, it would be important to replicate the present study in a clinical sample (e.g. generalised anxiety disorder; GAD) in order to test whether similar results emerge before offering any clinical conclusions. If so, various avenues for treatment could be explored in future research, testing possible explanations for the current findings. First, the additional use of longer SOAs would reveal if control resources are deployed at greater intervals after threat occurrence than those sampled here, and whether this can reduce longer term attentional bias effects. If so, this would support further work investigating whether control efforts can be made more effective; for example, by practice in deploying them as soon as possible after threat detection. Alternatively, varying the type of cognitive load or type of control measures would test if perceptual load is more effective in blocking bias assessed using visually presented threat cues (cf. Lavie et al. 2004; Pessoa et al. 2002).

However, worry involves attention to internal events such as negative intrusive thoughts so that a more critical test might involve testing the extent to which load blocks attention to negative thoughts (as opposed to visual attention to external cues). Previous research findings provide tentative evidence that intrusive negative thoughts can be reduced by substituting positive thoughts (Hirsch et al. 2015), although it remains unclear whether this effect is due to controlled or automatic processes. One approach to addressing this issue would be to contrast instructed (i.e. controlled) use of thought substitution with repeated practice aimed at increasing automatic access to alternative non-worry content.

In conclusion, we confirmed that high worriers are more likely to attend threat cues than low worriers, and further established that this bias persisted regardless of additional cognitive load, or reported attentional control ability. The latter findings provide no support for the hypothesis that biased attention to threat is dependent on controlled effort, either directed towards (vigilance) or away from threat cues (avoidance). Rather, the results are consistent with the possibility that biased attention towards threat cues in high worriers operates without deliberate effort and thus may be at least partially automatic. However, future research is required to test whether these findings depend on type of load or exposure duration: for example, more evidence of intentional efforts to avoid, or to maintain attention to threats, may emerge at longer exposure durations when controlled processes become more dominant. Nonetheless, the present results do provide support for the use of treatment approaches designed to modify early and relatively automatic attention processes, which may contribute to the onset of uncontrollable worry episodes (Hirsch and Mathews 2012).

## Footnotes

1 Separate 2 × 2 × 2 mixed-design repeated-measures ANOVAs were run for each mood rating (anxious, depressed, and happy). The anxiety ratings reported a significant main effect of group, with high worriers being more anxious than low worriers overall [*F*(1,50) = 59.85, *p* < .001; High *M* = 58.29, *SD* = 16.43; Low *M* = 19.38, *SD* = 19.69]; and a main effect of time, demonstrating a significant increase in anxiety between before worry inductions and after worry inductions [*F*(1,50) = 14.88, *p* < .001; Pre *M* = 35.79, *SD* = 25.48; Post *M* = 41.88, *SD* = 28.89]. There were no significant two-way or three-way interactions. A similar opposite pattern of results occurred for the happiness ratings as expected. Depression ratings reported a significant main effect of group, with high worriers being significantly more depressed than low worriers overall [*F*(1,50) = 29.65, *p* < .001; High *M* = 33.54, *SD* = 18.20; Low *M* = 9.84, *SD* = 12.70]; and a main effect of time, demonstrating a significant increase in depression ratings between before worry inductions and after worry inductions [*F*(1,50) = 49.49, *p* < .001; Pre *M* = 17.91, *SD* = 17.88; Post *M* = 25.46, *SD* = 22.18]. There was also a significant group x time interaction [*F*(1,50) = 17.60, *p* < .001], with high worriers reporting a greater increase in depression ratings pre- to post-worry induction (*M* difference = 12.2) than low worriers (*M* difference = 2.9). There were no other significant two-way or three-way interactions.
